# Supplementary material for: Natural variation of Dt2 determines branching in soybean
Source: Nat Commun. 2022 Oct 28;13:6429. doi: 10.1038/s41467-022-34153-4 (PMC9616897; doi:10.1038/s41467-022-34153-4)
Supplement: Supplementary file 1 — Supplementary Information [file 41467_2022_34153_MOESM1_ESM.pdf]

Natural variation of *Dt2* determines branching in soybean

Liang *et al.*

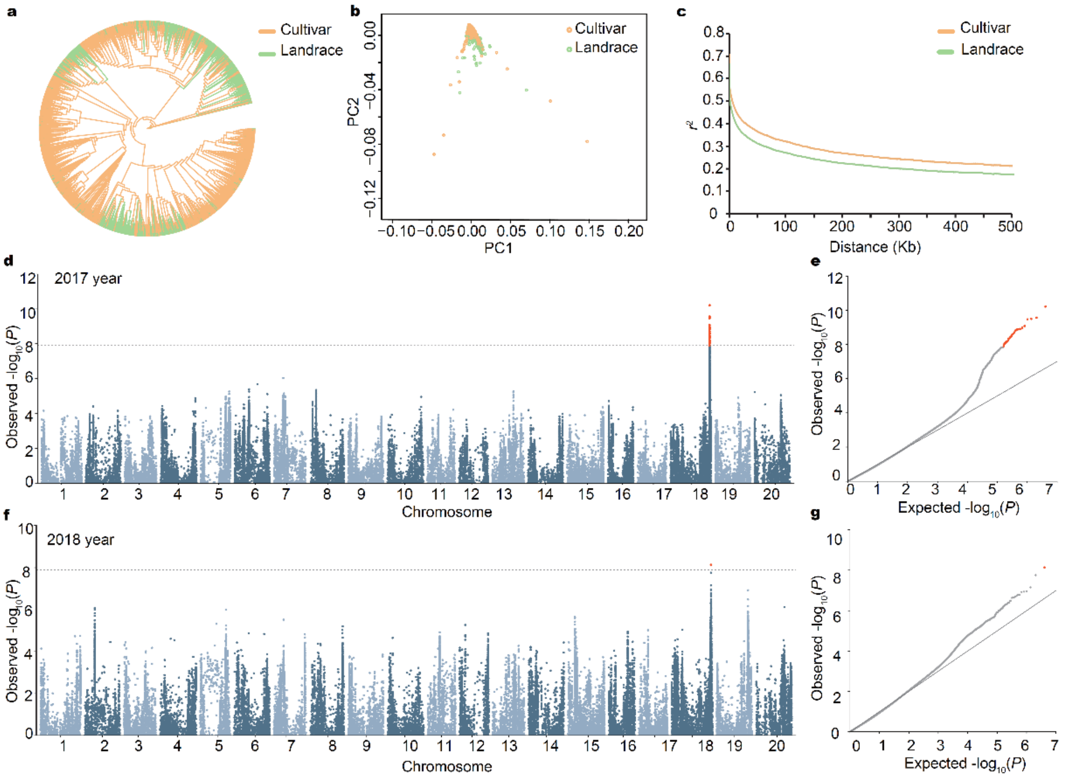

Supplementary Fig. 1. Natural population structure and GWAS of branch number. **a**, Phylogenetic structure analysis for the natural population. **b**, PCA plots of the first two components of the natural population. **c**, Decay of linkage disequilibrium (LD) in landraces (green), and cultivars (orange). LD decay is calculated based on the squared correlations of allele frequencies (r^2) against the distance between polymorphic sites. **d-g**, GWAS scan for branch number (R4 stage) using data from the 2,409 accession panel over the 2017 field season in Tianjin (**d-e**) and the 2018 field season in Beijing (**f-g**), China. In **d-g**, *P* values are calculated based on linear mixed model in GWAS and the dashed horizontal line indicates the genome-wide significance threshold ($P = 1 \times 10^{-7.9}$) , which is determined by the Bonferroni test. $-\log_{10} P$ values are plotted against the position of SNPs on 20 chromosomes. The red lead SNPs are shown above the threshold signals. For quantile–quantile plot, $-\log_{10}$-transformed observed *P* values are plotted against $-\log_{10}$-transformed expected *P* values. Source data are provided as a Source Data file.

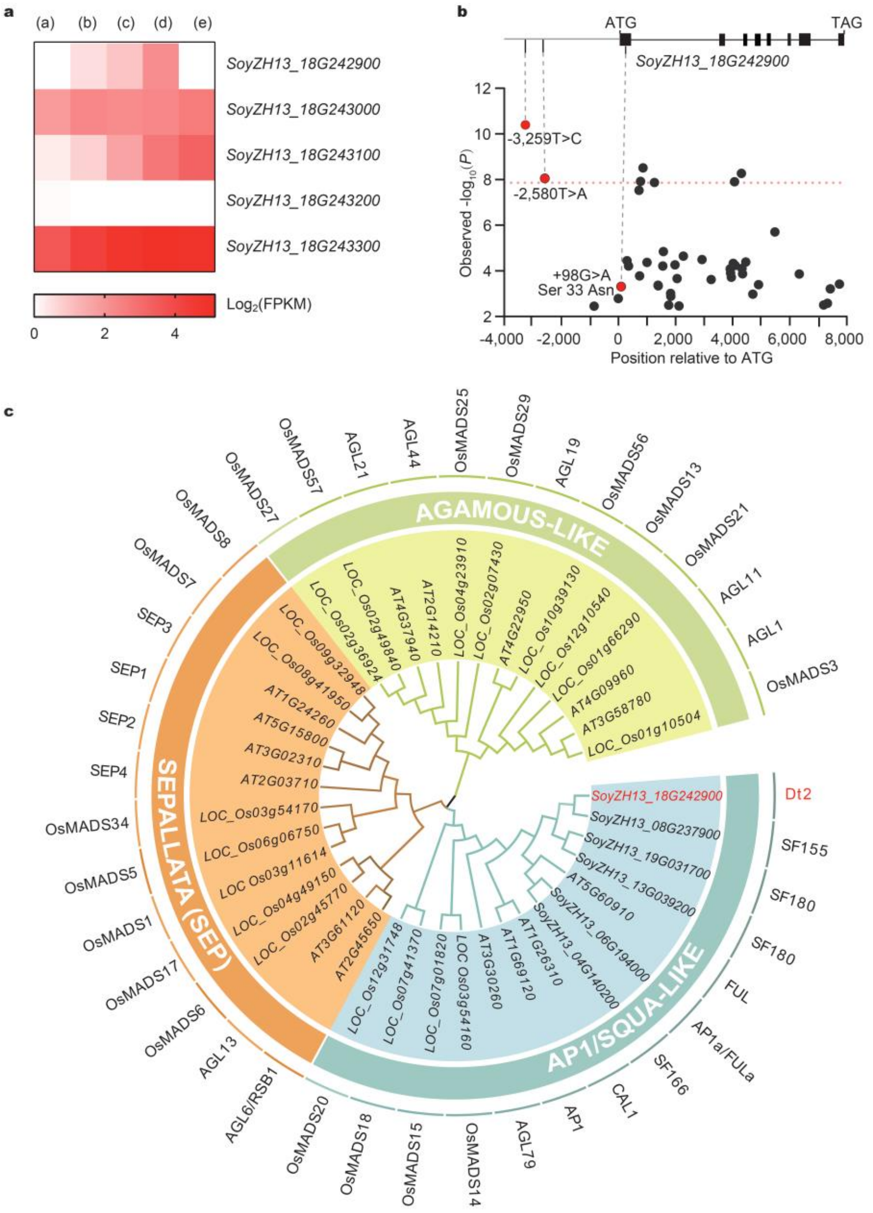

Supplementary Fig. 2. Candidate gene identification from GWAS results. a, Expression levels of 5 candidate genes in different tissues. (a)-(c) represent the leaf buds of VC, V1, and V2, respectively. (d)-(e) represent shoot apical meristem and flower, respectively. **b**, Association SNP structure analysis in the gene body and promoter region of *Dt2*. The red circle denotes SNP57808962 (-3,259 T>C), SNP57809641 (-2,580 T>A), and SNP57812381 (+98 G>A). Other black dots represent SNPs with low haplotype frequency (< 10%) or located in the intron. *x* axis, position relative to ATG (0 bp). *P* values are calculated based on linear mixed model in GWAS and the significance threshold determined by the Bonferroni test. **c**, Phylogenetic tree of homologous genes of *Dt2* among soybean, rice and *Arabidopsis*. The tree is constructed by the neighbor-joining method in MEGA 6.0 and the bootstrap is 1,000 replicates. Source data are provided as a Source Data file.

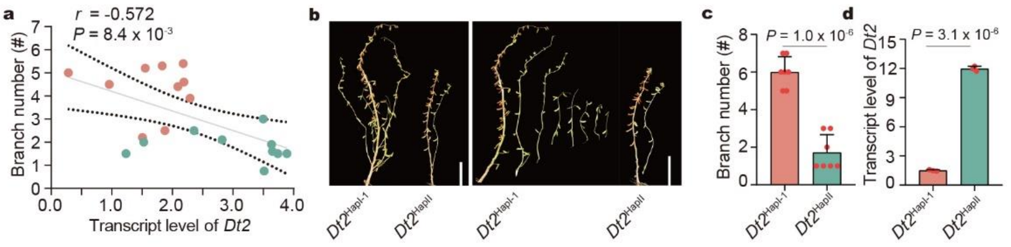

Supplementary Fig. 3. The promoter of *Dt2* affects its expression level. a, Correlation between the expression level of *Dt2* and branch number among the random *Dt2*$^{\mathrm{HapI\text{-}1}}$ and *Dt2*^{HapII} materials. The pink dots represent the *Dt2*^{HapII} material and the navy blue dots represent the *Dt2*$^{\mathrm{HapI\text{-}1}}$ material. $P < 0.05$ is the significant two-tailed value that analyzed by the Pearson correlation test, r is the correlation index. **b-c**, Phenotypic comparison between *Dt2*$^{\mathrm{HapI\text{-}1}}$ and *Dt2*^{HapII} near isogenic lines (NILs) (n = 7 biologically independent plants). Scale bars, 20 cm. **d**, *Dt2* expression level differences in *Dt2*$^{\mathrm{HapI\text{-}1}}$ and *Dt2*^{HapII} NILs (n = 3 biologically independent replicates). Data in **c** and **d** are mean ± SEM. Significant P value is $P < 0.05$, two sided t test. Source data are provided as a Source Data file.

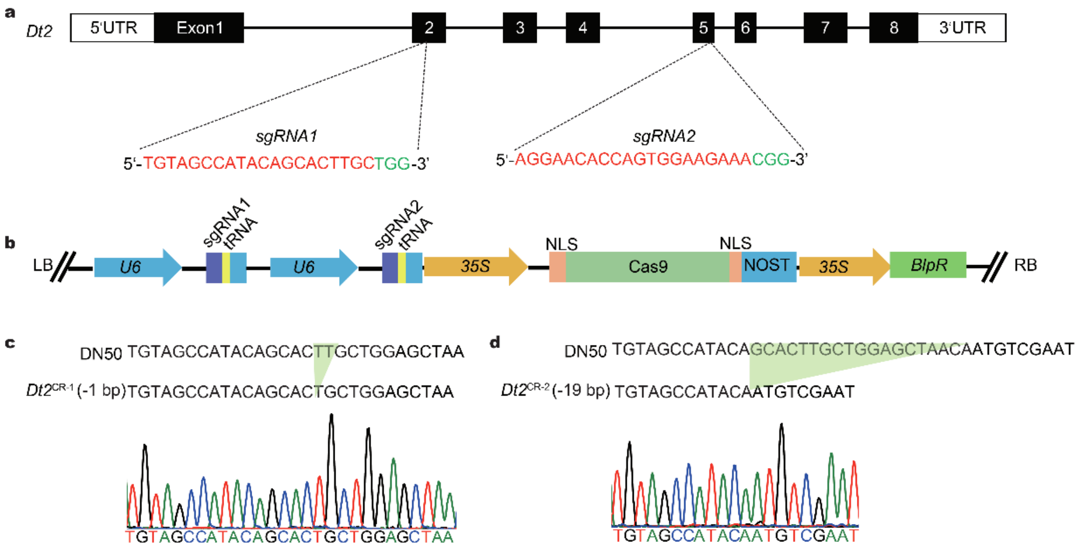

Supplementary Fig. 4. *Dt2* knockout mutant line creation in the CRISPR/Cas9 system. a, Target sites position design of *Dt2* are used for the CRISPR/Cas9 genome editing system. The red sequence indicates a targeted sequence. **b**, Partial vector map of PMDC123 in the CRISPR/Cas9 system. **c-d**, Two different edited events, *Dt2*$^{CR-1}$ (-1 bp) and *Dt2*$^{CR-2}$ (-19 bp), are generated by CRISPR/Cas9 in T_2 plants. The edited sequences are labeled with cyan triangles.

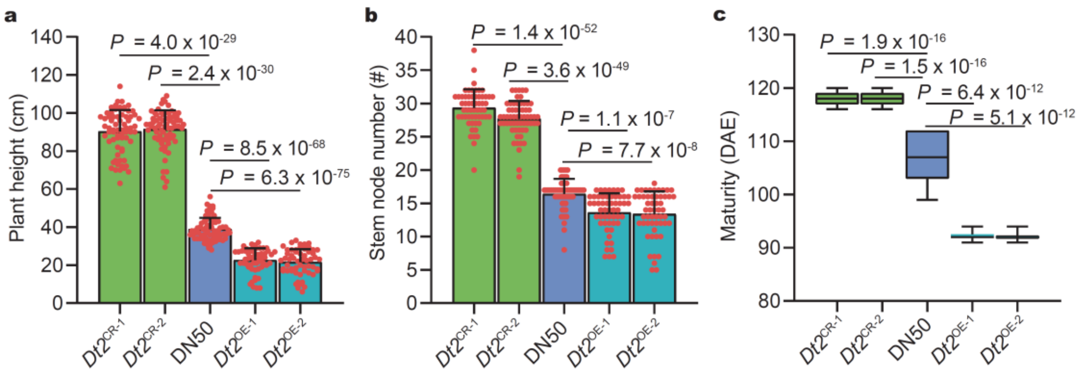

Supplementary Fig. 5. Phenotypic characteristics of *Dt2* transgenic lines in the field. a, Plant height of DN50, *Dt2*^{CR}, and *Dt2*^{OE} lines (n = 70 biologically independent plants). **b**, Stem node number of DN50, *Dt2*^{CR}, and *Dt2*^{OE} lines (n = 70 biologically independent plants). **c**, Maturity of DN50, *Dt2*^{CR}, and *Dt2*^{OE} lines (n = 30 biologically independent plants). In each box plot (drawn by R 4.1.1 software), the center line indicates the median, the edges of the box represent the first and third quartiles, and the whiskers extend to span a 1.5 interquartile range from the edges. Data in **a-c** are mean ± SEM. Statistical significance is determined using a two-sided *t*-test. Source data are provided as a Source Data file.

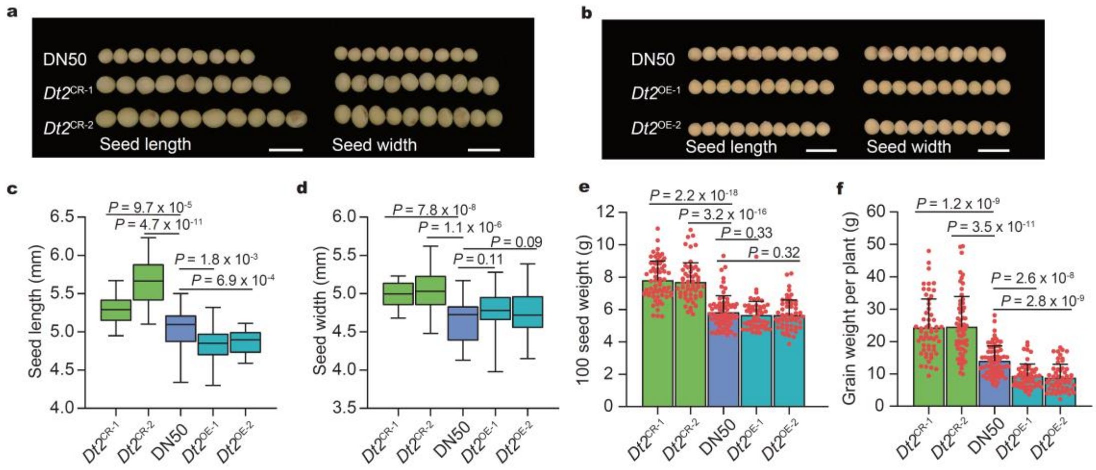

Supplementary Fig. 6. Yield trait characteristics of *Dt2* transgenic lines. a, Seed length and seed width comparison between DN50 and *Dt2*^{CR} lines (*n* = 30 biologically independent plants). Scale bars, 1 cm. **b**, Seed length and seed width comparison between DN50 and *Dt2*^{OE} lines (*n* = 30 biologically independent plants). Scale bars, 1 cm. **c-f**, Seed length and seed width, 100 seed weight (*n* = 70 biologically independent plants), grain weight per plant (*n* = 70 biologically independent plants) comparison among the DN50, *Dt2*^{CR} and *Dt2*^{OE} lines. In all the box plots, the center line indicates the median, the edges of the box represent the first and third quartiles, and the whiskers extend to span a 1.5 interquartile range from the edges. Data in **c-f** represent the mean ± SEM. Statistical significance is determined using a two-sided *t*-test. Source data are provided as a Source Data file.

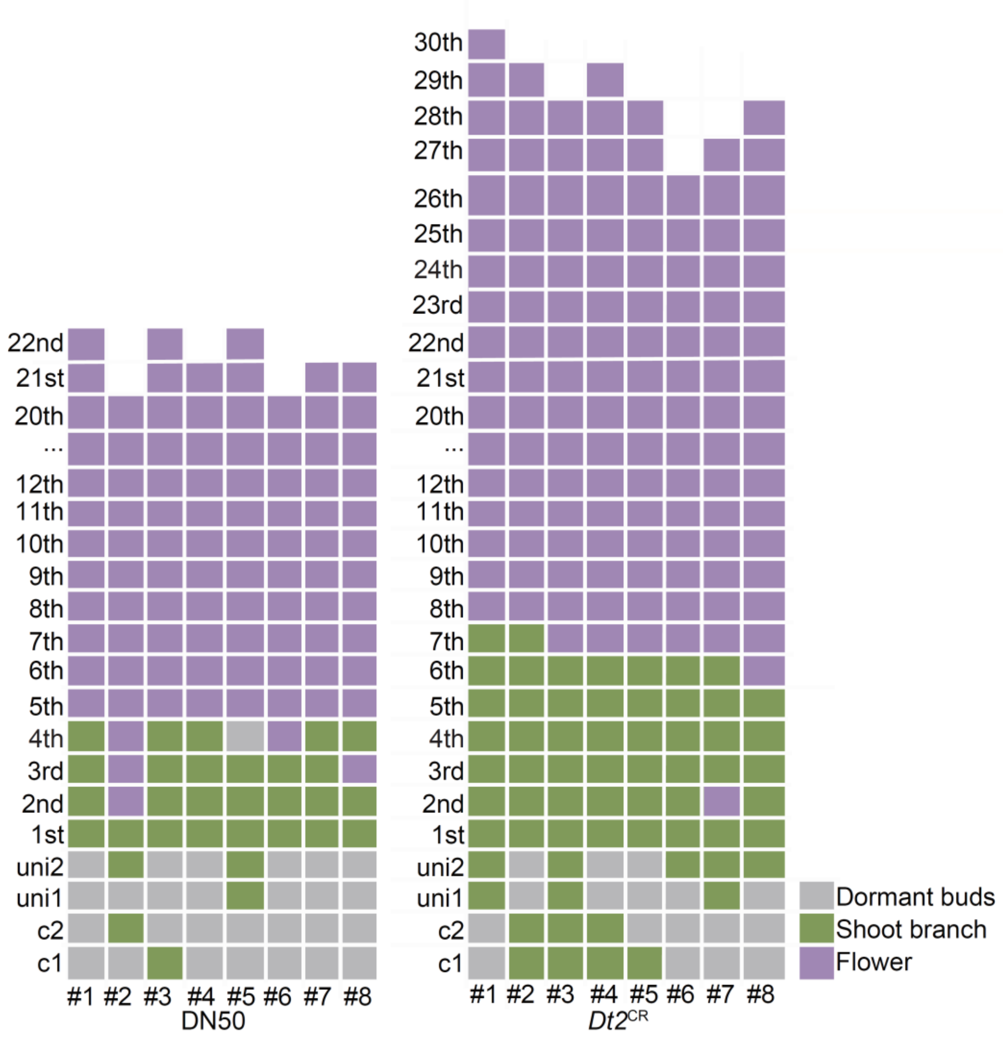

Supplementary Fig. 7. Growth state statistics of axillary buds in leaf axil between DN50 and *Dt2*^{CR} lines. Eight independent plants' axillary buds in leaf axils are collected from the cotyledon to apex node in the whole growth stage. c1 and c2 are the cotyledons axils, and uni1 and uni2 are the unifoliate leaves axils, 1st to 30th are the corresponding trifoliolate leaves axils.

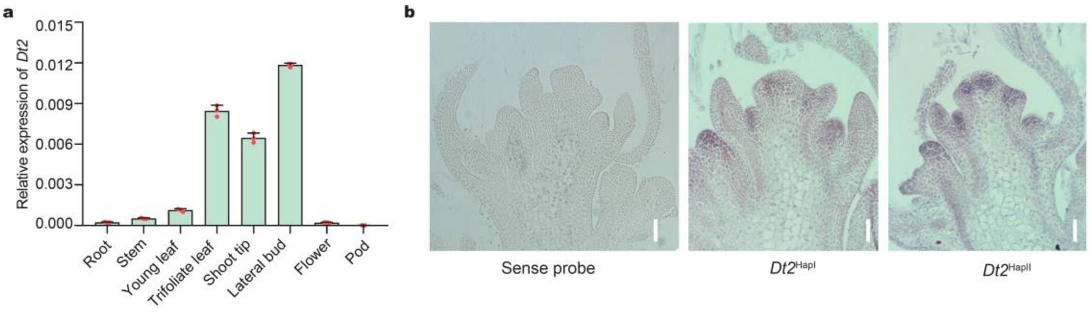

Supplementary Fig. 8. Expression pattern analysis of *Dt2*. a, Expression pattern analysis of *Dt2* in different developmental tissues of DN50 (n = 3 biologically independent replicates). Data are the mean ± SEM. **b**, In situ hybridization of *Dt2*. The figures show longitudinal sections of the SAM at the 16th day after emergence (DAE). At least 5 independent samples are embedded and a representative result is shown. Scale bars, 10 μm in the sense probe, 20 μm in the $Dt2^{HapI}$ and $Dt2^{HapII}$ figures. Source data are provided as a Source Data file.

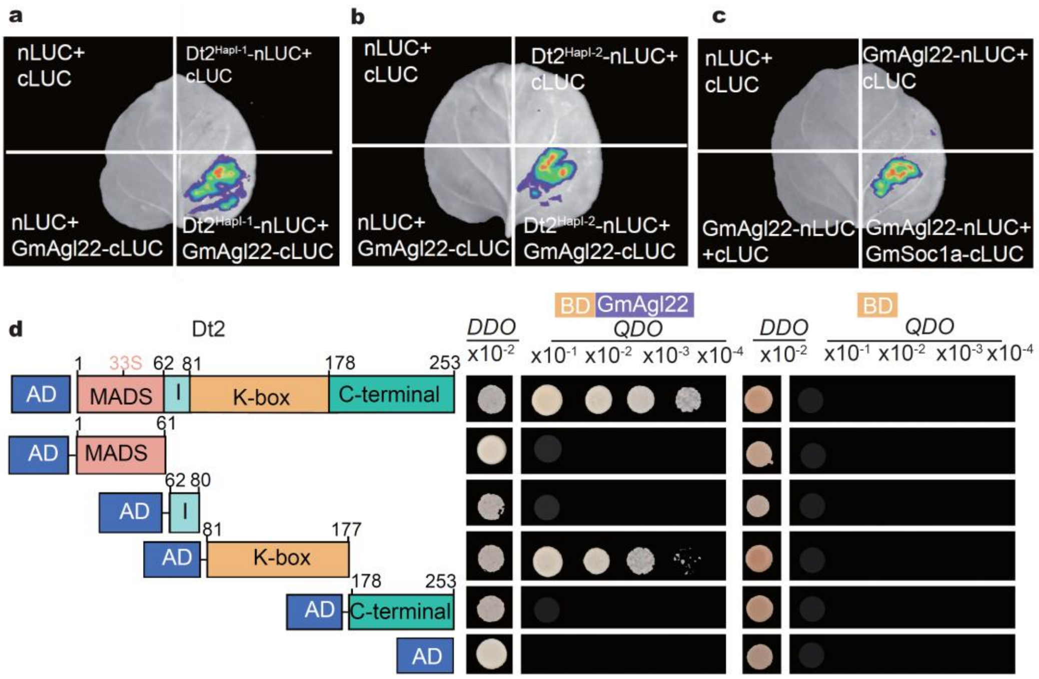

Supplementary Fig. 9. Split luciferase complementation assay among Dt2, GmAgl22 and GmSoc1a and dissection assay of Dt2. **a**, Split luciferase complementation assay between Dt2^{HapI-1} and GmAgl22. **b**, Split luciferase complementation assay between Dt2^{HapI-2} and GmAgl22. **c**, Split luciferase complementation assay between GmAgl22 and GmSoc1a. **d**, Dissection of the essential domain in Dt2 for the interaction between Dt2 and GmAgl22. In **a**-**c**, at least 5 independent leaves are transformed and a representative result is shown.

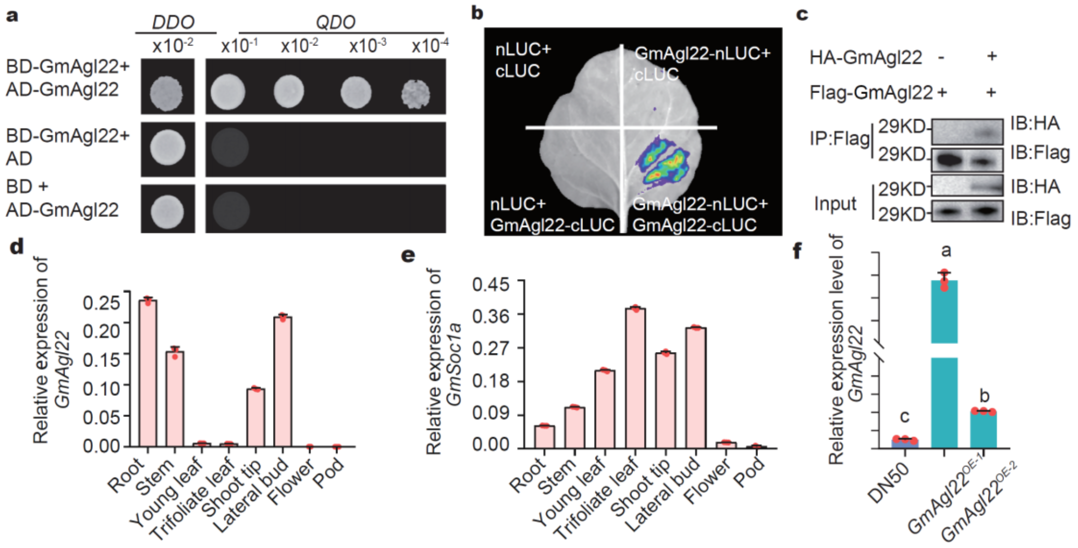

Supplementary Fig. 10. Molecular characteristics of *GmAgl22*. a, Interaction between GmAgl22 and GmAgl22 in the yeast two-hybrid assay. Transformed yeast cells are grown on *DDO* (Trp/Leu) or *QDO* (Trp/Leu/His/Ade) synthetic dropout medium. The numbers at the top indicate four serial dilutions. **b**, Luciferase complementation assay between GmAgl22 and GmAgl22. At least 5 independent leaves are transformed and a representative result is shown. **c**, Co-IP analysis of the protein interactions between GmAgl22 and GmAgl22. Flag-tagged GmAgl22 is co-transformed with HA-tagged GmAgl22 into *Arabidopsis* protoplasts. At least 3 independent replicates are performed and a representative result is shown. **d-e**, Expression pattern analysis of *GmAgl22* and *GmSoc1a* in different developmental tissues of DN50 ($n = 3$ biologically independent replicates). **f**, Relative expression level analysis of *GmAgl22* in DN50 and *GmAgl22*^{OE} lines. Different letters indicate statistically significant differences at $P < 0.05$ by one-way ANOVA test ($n = 3$ biologically independent replicates). Data in **d-f** are the mean ± SEM. At least 5 independent samples are embedded and a representative result is shown. Source data are provided as a Source Data file.

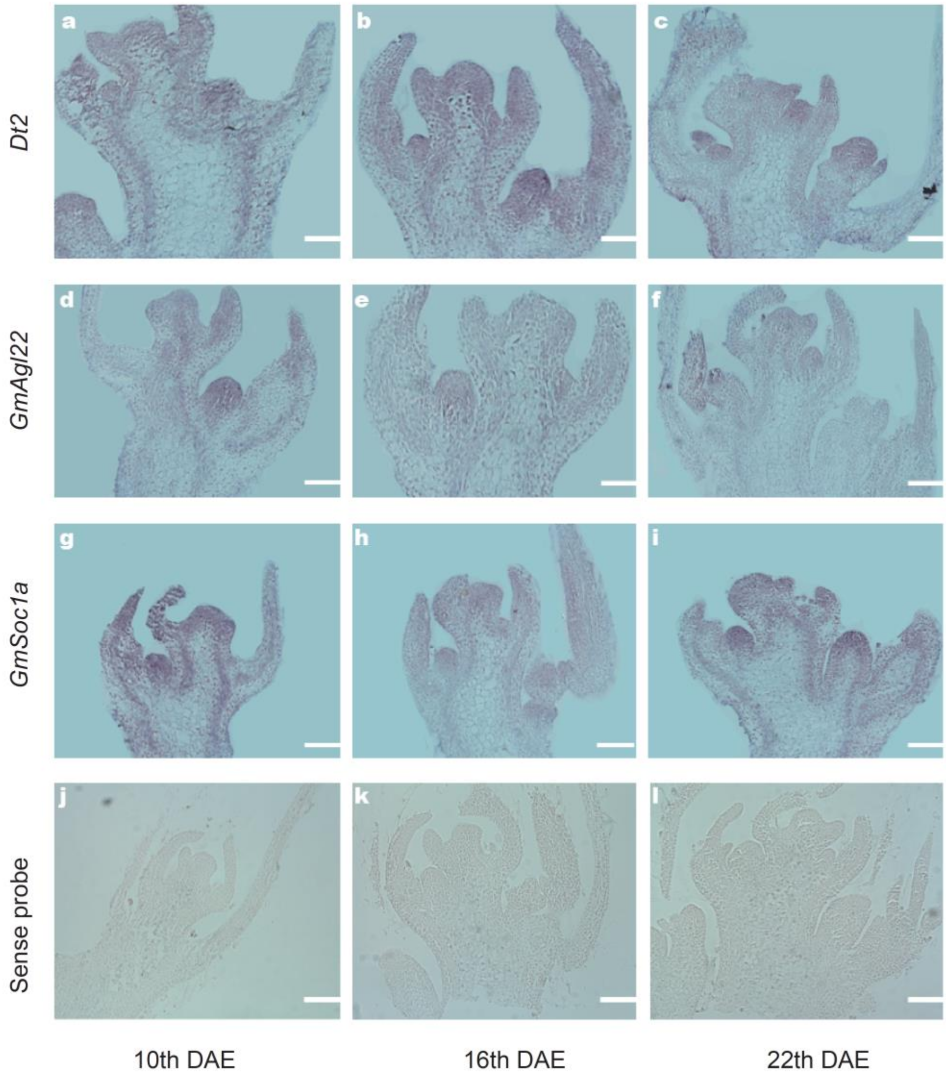

Supplementary Fig. 11. The spatio-temporal expression pattern analysis of *Dt2*, *GmAgl22* and *GmSoc1a* in SAM in different developmental stages of DN50. a-c, In situ hybridization assay of *Dt2* in the 10th, 16th and 22nd DAE of SAM in DN50. **d-f**, In situ hybridization assay of *GmAgl22* in the 10th, 16th and 22nd DAE of SAM in DN50. **g-i**, In situ hybridization assay of *GmSoc1a* in the 10th, 16th and 22nd DAE of SAM in DN50. **j-l**, The sense probe of *Dt2*, *GmAgl22*, *GmSoc1a* in situ hybridization assay in different stages of SAM of DN50 material. DAE indicates days after emergence. All the scale bars are 10 μm, and at least 5 independent samples are embedded and a representative result is shown.

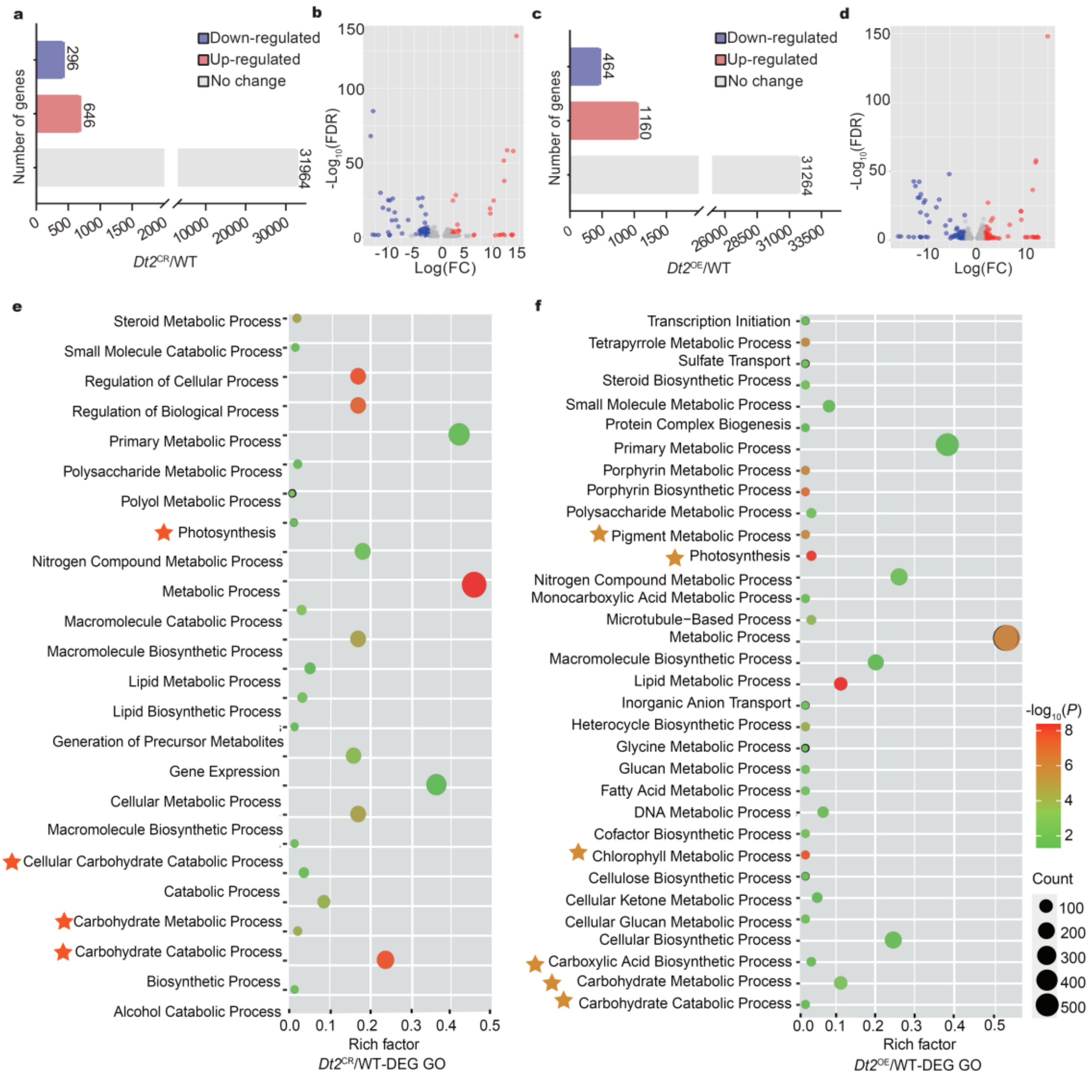

Supplementary Fig. 12. Analysis of the differentially expressed genes (DEGs). a, DEG analysis of *Dt2*^{CR} material versus the wild type DN50 (*Dt2*^{CR}/WT). **b**, The volcano plot of DEGs in *Dt2*^{CR}/WT. **c**, DEG analysis of *Dt2*^{OE} mutants versus the wild type DN50 (*Dt2*^{OE}/WT). **d**, The volcano plot of DEGs in *Dt2*^{OE}/WT. **e**, GO analysis of DEGs in *Dt2*^{CR}/WT. **f**, GO analysis of DEGs in *Dt2*^{OE}/WT. The rich factor is the ratio of query items to background items (query items/bgitem). *P* values are calculated in the online website agriGo (http://bioinfo.cau.edu.cn/agriGO/index.php). Count is the gene number in GO items. The pentagram represents the GO terms of interest, which mainly included photosynthesis and carbon metabolism processes.

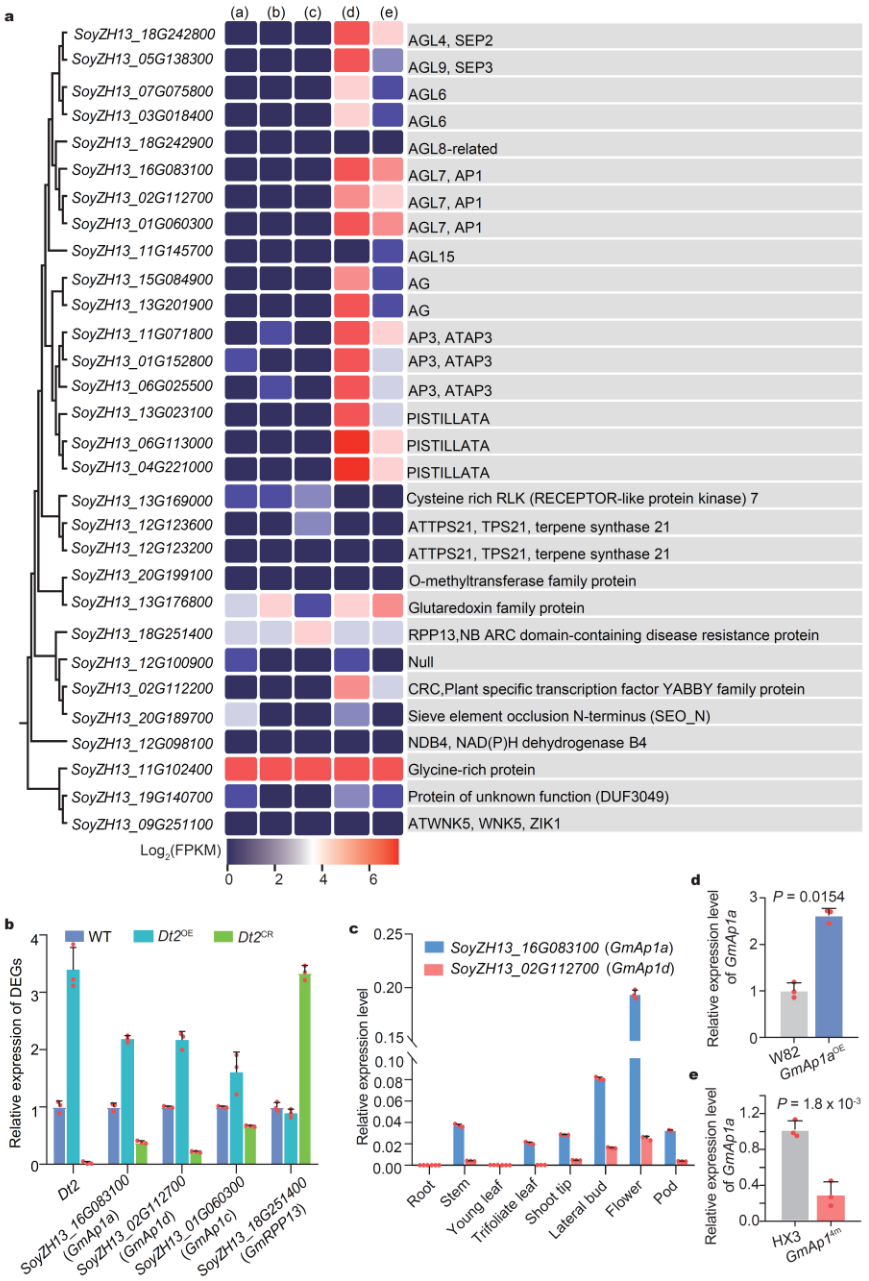

Supplementary Fig. 13. DEGs annotation and transcriptional analysis. a, Phylogenetic tree, expression pattern and corresponding functional annotation of 30 DEGs. (a)-(c), Leaf buds at the VC, V1, and V2 stages. (d)-(e), Flower and shoot apical meristem in the V2 stage. **b**, RT-qPCR analysis of *Dt2* and the four intersecting genes in different transgenic lines. **c**, Expression pattern analysis of *GmAp1a* and *GmAp1d* in different developmental stages of DN50. **d**, Relative expression level analysis of *GmAp1a* in W82 and *GmAp1a*^{OE} lines. **e**, Relative expression level analysis of *GmAp1a* in HX3 and *GmAp1a*^{4m} mutation lines. Data in **b-e** are the mean ± SEM. And $n = 3$ biologically independent replicates. Statistical significance is determined using a two-sided *t*-test. Source data are provided as a Source Data file.

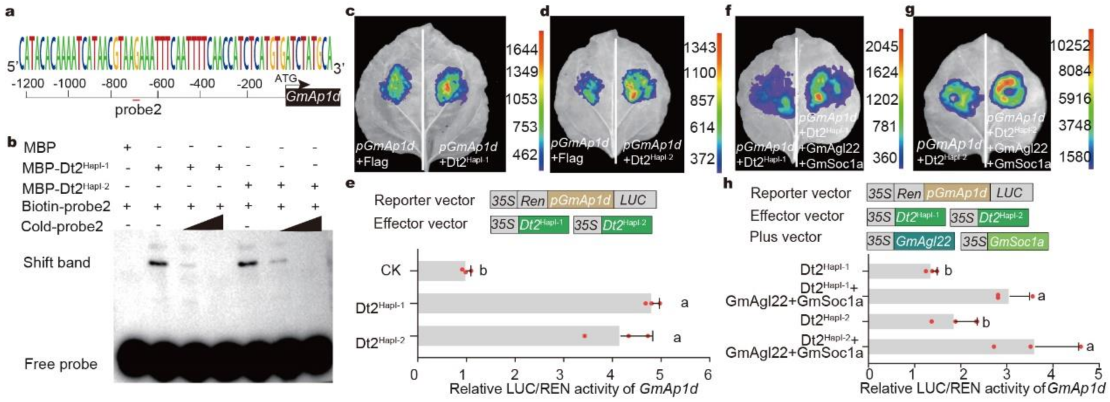

Supplementary Fig. 14. Dt2 directly binds the promoter of *GmAp1d* and positively regulates its expression. a, A probe sequence that could be bound by the MADS-box protein in the promoter of *GmAp1d* is predicted in Plantpan (http://plantpan.itps.ncku.edu.tw/). **b**, Dt2^{HapI-1} or Dt2^{HapI-2} protein directly bind to the *GmAp1d* promoter in EMSA. At least 3 independent replicates are performed and a representative result is shown. **c-d**, Transient dual luciferase (dual-LUC) assays of the transcriptional activity of Dt2 on the promoter of *GmAp1d* in tobacco leaves. **e**, Transient dual luciferase (dual-LUC) assay of Dt2 on the promoter of *GmAp1d* in *Arabidopsis* protoplasts. **f-g**, Dual-LUC assay in tobacco leaves of Dt2^{HapI-1} (**f**) or Dt2^{HapI-2} (**g**) and its interacting proteins GmAgl22 and GmSoc1a on the promoter of *GmAp1d*. **h**, Dual-LUC assay of Dt2 and its interacting proteins GmAgl22 and GmSoc1a on the promoter of *GmAp1d* in *Arabidopsis* protoplasts. Data in **e** and **h** are the mean ± SEM. Different letters indicate statistically significant differences at $P < 0.05$ by one-way ANOVA test and $n = 3$ biologically independent replicates. Source data are provided as a Source Data file.

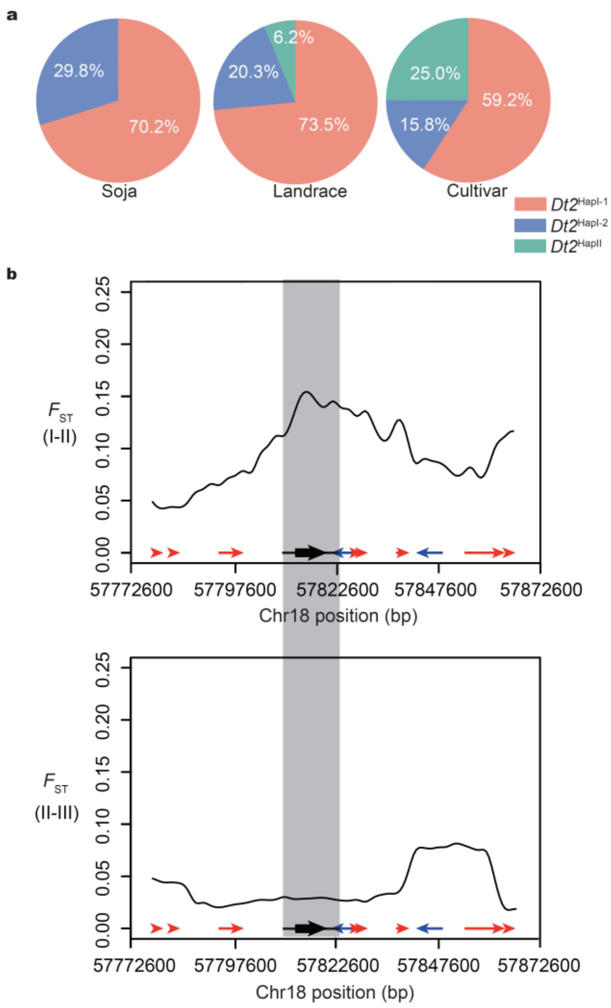

Supplementary Fig. 15. Genetic differentiation of *Dt2* in different material types and ecoregions in China. a, *Dt2* different haplotype percentage statistics among different material types. **b**, F_{ST} value detection in ecoregions I-II and II-III accessions across the 100 kb genomic region containing the *Dt2* locus. The gray region denote the *Dt2* gene and 3 kb region around its upstream and downstream regions.

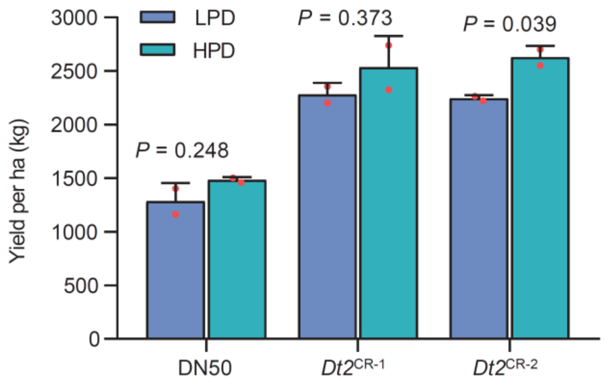

Supplementary Fig. 16. Effect of *Dt2* and planting densities on soybean yield. The average yields of DN50 and *Dt2*^{CR} lines in Beijing under low and high planting densities. Ha, hectare. LPD, low planting density (1,320 plants/100 m^2). HPD, high planting density (2,000 plants/100 m^2). The low and high density plots are 2 biologically independent replicates. Data are the mean ± SEM. Statistical significance is determined using a two-sided *t*-test. Source data are provided as a Source Data file.

Supplementary Table 1. The list of interacting proteins of Dt2.

Gene	No.[a]	*Arab.*[b]	Annotation
SoyZH13_17G097500	26	*AT3G47490*	HNH endonuclease
SoyZH13_02G039700	22	*AT2G22540*	AGL22, K-box region and MADS-box transcription factor family protein
SoyZH13_12G024100	20	*AT2G16600*	Rotamase CYP 3
SoyZH13_18G028700	14	*AT2G39990*	Eukaryotic translation initiation factor 2
SoyZH13_03G092600	10	*AT2G05170*	Vacuolar protein sorting 11
SoyZH13_10G205400	7	*AT1G71695*	Peroxidase superfamily protein
SoyZH13_03G184400	4	*AT2G35900*	Unknow
SoyZH13_16G039500	4	*AT3G04070*	NAC domain containing protein 47
SoyZH13_06G072500	3	*AT5G10700*	Peptidyl-tRNA hydrolase II (PTH2) family protein
SoyZH13_13G135100	3	*AT3G10070*	TBP-associated factor 12
SoyZH13_15G100800	2	*AT1G03220*	Eukaryotic aspartyl protease family protein
SoyZH13_09G038100	2	*AT5G49230*	HRB1,drought-responsive family protein
SoyZH13_14G099000	2	*AT2G24970*	Unknow
SoyZH13_06G154000	2	*AT4G38620*	ATMYB4,MYB4;myb domain protein 4
SoyZH13_20G112000	2	*AT2G01290*	Ribose-5-phosphate isomerase 2
SoyZH13_02G194100	1	*AT4G24620*	Phosphoglucose isomerase 1
SoyZH13_04G201500	1	*AT3G54190*	Transducin/WD40 repeat-like superfamily protein
SoyZH13_04G217800	1	*AT1G43850*	SEUSS transcriptional co-regulator
SoyZH13_06G071600	1	*AT1G71230*	COP9 signalosome complex subunit 5
SoyZH13_06G173800	1	*AT1G27500*	Tetratricopeptide repeat (TPR)-like superfamily protein
SoyZH13_07G238500	1	*AT2G36770*	UDP-Glycosyltransferase superfamily protein
SoyZH13_10G067000	1	*AT3G10070*	TBP-associated factor 12
SoyZH13_10G269100	1	*AT2G39420*	Alpha/beta-Hydrolases superfamily protein
SoyZH13_14G189100	1	*AT2G30520*	Phototropic-responsive NPH3 family protein
SoyZH13_16G146600	1	*AT1G34000*	One-helix protein 2
SoyZH13_18G206700	1	*AT3G61440*	Cysteine synthase 1
SoyZH13_19G018200	1	*AT5G62740*	PHB domain-containing membrane-associated protein family

[a] The number of yeast clone. [b] the homologous gene in *Arabidopsis*.

Supplementary Table 2. The number of *Dt2*^{HapI} and *Dt2*^{HapII} accessions in three different ecoregions.

Ecoregions	Type	*Dt2*^{HapI}	*Dt2*^{HapII}	Percentage % (*Dt2*^{HapII})
I	Landrace	151	28	15.64
	Cultivar	253	263	50.97
	Total	404	291	41.87
II	Landrace	293	13	4.25
	Cultivar	462	53	10.29
	Total	755	66	8.04
III	Landrace	127	1	0.78
	Cultivar	94	0	0.00
	Total	221	1	0.45

Supplementary Table 3. The average yield of DN50 and *Dt2*^{CR} lines in three latitude locations.

Latitude location	Materials	Repeat1	Repeat2	Average yield (kg/ha)	Increased ratio (%)*
I	DN50	2176	2276	2226	-
	Dt2^{CR-1}	3337	3108	3223	44.8
(Heilongjiang)	*Dt2*$^{CR-2}$	3558	3317	3438	54.4
II	DN50	1405	1163	1284	-
	Dt2^{CR-1}	2357	2204	2281	77.7
(Beijing)	*Dt2*$^{CR-2}$	2221	2266	2243	74.7
III	DN50	343	362	352	-
	Dt2^{CR-1}	665	514	590	67.5
(Hainan)	*Dt2*$^{CR-2}$	497	480	489	38.9

Note: * The ratio is the average of *Dt2*^{CR} lines compared with DN50.
