# Supplementary material for: Natural variation of Dt2 determines branching in soybean
Source: Nat Commun. 2022 Oct 28;13:6429. doi: 10.1038/s41467-022-34153-4 (PMC9616897; doi:10.1038/s41467-022-34153-4)
Supplement: Supplementary file 3 — Description of Additional Supplementary Files [file 41467_2022_34153_MOESM3_ESM.pdf]

Description of Additional Supplementary Files

File Name: **Supplementary Data 1**
Description: Geographic information of soybean accessions in China.

File Name: **Supplementary Data 2**
Description: The primer sequences used in this study.
