# Supplementary material for: Natural variation of Dt2 determines branching in soybean
Source: Nat Commun. 2022 Oct 28;13:6429. doi: 10.1038/s41467-022-34153-4 (PMC9616897; doi:10.1038/s41467-022-34153-4)
Supplement: Supplementary file 6 — Reporting Summary [file 41467_2022_34153_MOESM6_ESM.pdf]

nature portfolio

Corresponding author(s): Zhixi Tian, NCOMMS-22-20962

Last updated by author(s): Oct 10, 2022

Reporting Summary

Nature Portfolio wishes to improve the reproducibility of the work that we publish. This form provides structure for consistency and transparency in reporting. For further information on Nature Portfolio policies, see our Editorial Policies and the Editorial Policy Checklist.

Statistics

For all statistical analyses, confirm that the following items are present in the figure legend, table legend, main text, or Methods section.

n/a	Confirmed	
☐	☒	The exact sample size (n) for each experimental group/condition, given as a discrete number and unit of measurement
☐	☒	A statement on whether measurements were taken from distinct samples or whether the same sample was measured repeatedly
☐	☒	The statistical test(s) used AND whether they are one- or two-sided<br>*Only common tests should be described solely by name; describe more complex techniques in the Methods section.*
☒	☐	A description of all covariates tested
☒	☐	A description of any assumptions or corrections, such as tests of normality and adjustment for multiple comparisons
☐	☒	A full description of the statistical parameters including central tendency (e.g. means) or other basic estimates (e.g. regression coefficient) AND variation (e.g. standard deviation) or associated estimates of uncertainty (e.g. confidence intervals)
☐	☒	For null hypothesis testing, the test statistic (e.g. F, t, r) with confidence intervals, effect sizes, degrees of freedom and P value noted<br>*Give P values as exact values whenever suitable.*
☒	☐	For Bayesian analysis, information on the choice of priors and Markov chain Monte Carlo settings
☒	☐	For hierarchical and complex designs, identification of the appropriate level for tests and full reporting of outcomes
☒	☐	Estimates of effect sizes (e.g. Cohen's d, Pearson's r), indicating how they were calculated

Our web collection on statistics for biologists contains articles on many of the points above.

Software and code

Policy information about availability of computer code

Data collection	The SNP data of 2,409 natural population accessions (Cell, 2020, 182,162-176) were uesed in this study.<br>The sequencing data for RNA-seq of 9 independently soybean samples were generated from the Illumina NovaSeq 2000 platform.
Data analysis	Phenotype data:<br>The phenotype data of natural population and transgenic lines were mean and t-test through the Microsoft excel 2016.<br>Best linear unbiased prediction (BLUP):<br>The BLUP data from the mean of the 2017 and 2018 natural population phenotype data and were calculated by lmer function from the R-lme4 (v1.1-30) library (https://github.com/lme4/lme4/).<br>GWAS analysis:<br>A total of 4,072,231 SNPs were used for association analysis with a minor allele frequency (MAF) of >5% and missing rate < 10%. GWAS was performed based on the efficient mixed-model association expedited (EMMAX) approach.<br>RNA-seq analysis:<br>The high-quality sequencing reads were mapped to the reference genome with Hisat (v. 2.2.1). And the gene expression counts were calculated using StringTie (v.1.3.4d). The different expression genes analysis were analyzed by the R-edgeR library (https://bioconductor.org/packages/release/bioc/html/edgeR.html).<br>Selection analysis:<br>The genetic differentiation fixation index (FST) was calculated by using VCFtools (0.1.13) with a 20 kb slide window and 2 kb slide step.

For manuscripts utilizing custom algorithms or software that are central to the research but not yet described in published literature, software must be made available to editors and reviewers. We strongly encourage code deposition in a community repository (e.g. GitHub). See the Nature Portfolio guidelines for submitting code & software for further information.

March 2021

Data

Policy information about availability of data

All manuscripts must include a data availability statement. This statement should provide the following information, where applicable:

- Accession codes, unique identifiers, or web links for publicly available datasets
- A description of any restrictions on data availability
- For clinical datasets or third party data, please ensure that the statement adheres to our policy

The SNP data of 2,409 natural population accessions were reported and have been deposited in the Genome Sequence Archive (GSA) database in the BIG Data Center (https://ngdc.cncb.ac.cn/) under accession number PRJNA257011, PRJNA394629 and CRA002269.
Raw sequencing data for RNA-seq in this study have been deposited in the Genome Sequence Archive (GSA) database in the BIG Data Center (https://ngdc.cncb.ac.cn/) under accession number SAMC797049-SAMC797057 of PRJCA009434.

Human research participants

Policy information about studies involving human research participants and Sex and Gender in Research.

Reporting on sex and gender	*Use the terms sex (biological attribute) and gender (shaped by social and cultural circumstances) carefully in order to avoid confusing both terms. Indicate if findings apply to only one sex or gender; describe whether sex and gender were considered in study design whether sex and/or gender was determined based on self-reporting or assigned and methods used. Provide in the source data disaggregated sex and gender data where this information has been collected, and consent has been obtained for sharing of individual-level data; provide overall numbers in this Reporting Summary. Please state if this information has not been collected. Report sex- and gender-based analyses where performed, justify reasons for lack of sex- and gender-based analysis.*
Population characteristics	*Describe the covariate-relevant population characteristics of the human research participants (e.g. age, genotypic information, past and current diagnosis and treatment categories). If you filled out the behavioural & social sciences study design questions and have nothing to add here, write "See above."*
Recruitment	*Describe how participants were recruited. Outline any potential self-selection bias or other biases that may be present and how these are likely to impact results.*
Ethics oversight	*Identify the organization(s) that approved the study protocol.*

Note that full information on the approval of the study protocol must also be provided in the manuscript.

Field-specific reporting

Please select the one below that is the best fit for your research. If you are not sure, read the appropriate sections before making your selection.

☒ Life sciences ☐ Behavioural & social sciences ☐ Ecological, evolutionary & environmental sciences

For a reference copy of the document with all sections, see nature.com/documents/nr-reporting-summary-flat.pdf

Life sciences study design

All studies must disclose on these points even when the disclosure is negative.

Sample size	The sample size was determined according to the reports in the related research subjects. For the field phenotype assay, 5 represent plant growing in unison of one accession were selected from 2,409 natural population in R4 stage, namely the full pod stage. For the phenotype statistic for the transgenic lines, at least 20 samples in full maturity were investigated in field for the students' t test analysis in determining the phenotype traits. For the genes' expression level quantification, 3 biological replicates were performed for each sample. For the Pearson correlation analysis, 20 materials' branch number (R4 stage) and correspond Dt2 expression level were analyzed. For RNA-seq experiment, about 100 mg of lateral buds were collected from the V2 stage's plant, and 3 independent repeats of every type materials. For the students' t test, at least 3 independent samples were required, so the number of samples were selected more than 3. No statistical methods were used to predetermine sample sizes.
Data exclusions	No data were excluded from our analysis.
Replication	For each experiment, at least three independently biological experiments were repeated.
Randomization	The experiments were used to compared the phenotype traits between transgenic lines and wild type, therefore, sample allocation is not relevant to this study.
Blinding	The experiments were used to compared the phenotype traits between transgenic lines and wild type, therefore, blinding is not relevant to this study.

Reporting for specific materials, systems and methods

We require information from authors about some types of materials, experimental systems and methods used in many studies. Here, indicate whether each material, system or method listed is relevant to your study. If you are not sure if a list item applies to your research, read the appropriate section before selecting a response.

Materials & experimental systems

n/a	Involved in the study
☐	☒ Antibodies
☒	☐ Eukaryotic cell lines
☒	☐ Palaeontology and archaeology
☒	☐ Animals and other organisms
☒	☐ Clinical data
☒	☐ Dual use research of concern

Methods

n/a	Involved in the study
☒	☐ ChIP-seq
☒	☐ Flow cytometry
☒	☐ MRI-based neuroimaging

Antibodies

Antibodies used

Anti-HA-tag, mAb-HRP-DirecT, MBL (lot: M180-7)
Anti-DDDDK-tag, mAb-HRP-DirecT, MBL (lot: M185-7)

Validation

The Anti-HA-tag (M180-7) antibody validation could be found in the website: http://www.mbl-chinawide.cn/search-details2?id=1529&table=RuoAntibody
And the Anti-DDDDK-tag (M185-7) antibody validation could be found in the website: http://www.mbl-chinawide.cn/search-details2?id=963&table=RuoAntibody
